# Deep-learning image analysis for high-throughput screening of opsono-phagocytosis-promoting monoclonal antibodies against *Neisseria gonorrhoeae*

**DOI:** 10.1038/s41598-024-55606-4

**Published:** 2024-02-27

**Authors:** Fabiola Vacca, Dario Cardamone, Emanuele Andreano, Duccio Medini, Rino Rappuoli, Claudia Sala

**Affiliations:** 1 Monoclonal Antibody Discovery Laboratory, Fondazione Toscana Life Sciences, Siena, Italy; 2 Data Science for Health Laboratory, Fondazione Toscana Life Sciences, Siena, Italy; 3 University of Turin, Turin, Italy; 4 Fondazione Biotecnopolo Di Siena, Siena, Italy

**Keywords:** Computational biology and bioinformatics, Microbiology

## Abstract

Antimicrobial resistance (AMR) is nowadays a global health concern as bacterial pathogens are increasingly developing resistance to antibiotics. Monoclonal antibodies (mAbs) represent a powerful tool for addressing AMR thanks to their high specificity for pathogenic bacteria which allows sparing the microbiota, kill bacteria through complement deposition, enhance phagocytosis or inhibit bacterial adhesion to epithelial cells. Here we describe a visual opsono-phagocytosis assay which relies on confocal microscopy to measure the impact of mAbs on phagocytosis of the bacterium *Neisseria gonorrhoea*e by macrophages. With respect to traditional CFU-based assays, generated images can be automatically analysed by convolutional neural networks. Our results demonstrate that confocal microscopy and deep learning-based analysis allow screening for phagocytosis-promoting mAbs against *N. gonorrhoeae*, even when mAbs are not purified and are expressed at low concentration. Ultimately, the flexibility of the staining protocol and of the deep-learning approach make the assay suitable for other bacterial species and cell lines where mAb activity needs to be investigated.

## Introduction

Antimicrobial resistance (AMR) refers primarily to the ability of bacteria to survive exposure to static or cidal antibiotics and represents a global public health concern^1^. As a result of AMR, standard treatments have become less effective, and infections are harder or impossible to control, thus increasing the risk of spreading morbidity and mortality^2,3^.

An innovative and promising strategy to fight AMR consists in exploiting human monoclonal antibodies (mAbs). Despite the difficulties in targeting bacterial pathogens, several mAb candidates are progressing through clinical development and more mAbs against Gram-negative AMR species are being discovered^4^. mAbs can be isolated directly from patients who survived a specific infection by means of the so-called Reverse Vaccinology 2.0 approach^5^. Key to this strategy is an effective high-throughput screening technology that allows fast identification of the most potent antibacterial mAbs among the many thousands produced by each patient.

mAbs exert different mechanisms of action when facing bacterial infections. They can trigger the complement cascade, induce agglutination of pathogens or of toxins or, in the presence of specialised immune cells like macrophages or neutrophils, enhance their intrinsic opsono-phagocytic activity^6^. Antibody-mediated phagocytosis is the process by which a pathogen is marked for ingestion and eliminated by a phagocyte. The Fab region of the antibody binds to the antigen, whereas the Fc region of the antibody binds to an Fc receptor on the phagocyte, thereby facilitating phagocytosis^7^. In the early stages of phagocytosis, antibodies enhance internalisation and engulfment of the opsonized bacteria by the macrophages. In the latest stages, antibodies can restrict the survival of engulfed bacterial pathogens which may have developed mechanisms of phagosomal escape^8^.

In this context, we focused our attention on *Neisseria gonorrhoeae*, the etiological agent of gonorrhoea, a sexually transmitted infection that affects more than 100 million people annually and has become a major threat given the steady increase in AMR isolates^9^. No vaccine against *N. gonorrhoeae* is currently available and development of preventive tools has proven complicated in the past few decades^10^. Among therapeutic anti-*N. gonorrhoeae* mAb candidates, an antibody directed against the gonococcal lipooligosaccharide (LOS), named 2C7, was developed. In their seminal work Gulati and colleagues reported that 2C7 mediated complement-dependent killing and phagocytosis of bacteria *in vitro*^11^.

The reference assay described in the literature to study phagocytosis of *N. gonorrhoeae* relies on colony forming unit (CFU) counting upon macrophage cell lysis^12^. However, CFU counting is a tedious and time-consuming process that cannot explore host–pathogen interaction at the single-cell level^13^. In an attempt to increase the throughput, Smirnov and co-workers employed the Fluorescence-Activated Cell Sorter (FACS) to measure internalisation of *N. gonorrhoeae* by neutrophils^14^.

**Figure 1:**
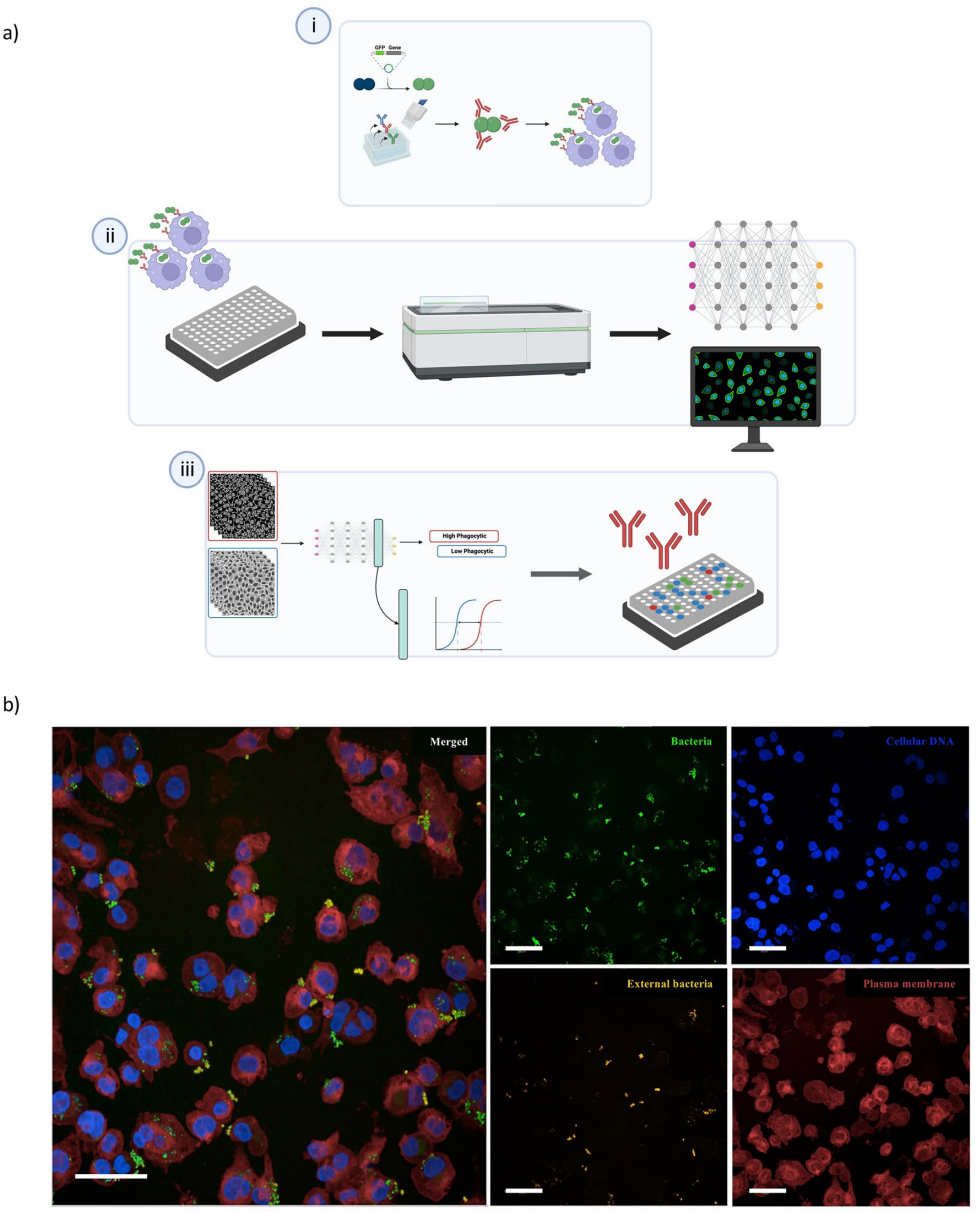
The vOPA workflow and staining used. (**a**) (i) *N. gonorrhoeae* is engineered to express GFP and mAbs are expressed as recombinant molecules. Bacteria are then incubated with the mAbs and used to infect differentiated THP-1 cells. (ii) Infection is conducted in a 96-well plate for fluorescence microscopy imaging. Following staining and fixation, images are acquired with the Opera Phenix microscope and (iii) analysed by fine-tuning a CNN model to classify experimental positive and negative controls. The obtained score is used to quantify the phagocytic activity of cells in presence or absence of mAbs. Ultimately, the most prominent candidates are selected. Image created with BioRender.com. (**b**) The large picture exemplifies images acquired at the Opera Phenix confocal microscope. The small pictures display the four channels in the vOPA assay protocol as imaged using the Opera Phenix microscope: sfGFP expressed by *N. gonorrhoeae* (green), DAPI to stain the nuclei and bacterial DNA (blue), Alexa 568 conjugated secondary antibody for immunostaining (orange) and CellMask Deep Red to stain cell membranes (red). Scale bar is 50 μm.

Here we demonstrate that high-throughput fluorescence microscopy and deep learning-based image analysis enabled the screening of several anti-*N. gonorrhoeae* mAb candidates for their opsono-phagocytosis-promoting activity. The experimental approach, which we named visual opsono-phagocytosis assay (vOPA), is depicted in Fig. 1a. First, *N. gonorrhoeae* strain FA1090 was engineered to constitutively express the Green Fluorescent Protein (GFP) (Fig. 1ai). Bacteria were then pre-incubated with mAbs and used to infect the monocyte cell line THP-1 in 96-well microplates for confocal imaging (Fig. 1ai). The confocal microscope Opera Phenix® High-Content Screening System allowed acquisition of images, which were then analysed by a deep neural network (Fig. 1aii). A Dense Convolutional Network (DenseNet^15^) was fine-tuned to classify previously validated positive and negative controls, while a linear Support Vector Machine (SVM) was exploited to screen and rank phagocytosis-promoting mAbs (Fig. 1aiii). Figure 1b shows a representative example of the staining used in vOPA, and the resulting images analysed to derive the Phagocytic score. DAPI stained cell nuclei and bacterial DNA, CellMask Deep Red stained cell membranes, GFP expressed by FA1090 marked the total bacteria population. To distinguish the internalised (engulfed) bacteria from the external bacteria, an immunostaining approach was employed. External bacteria were marked with a primary anti-gonococcal antibody followed by a secondary antibody, whereas internal bacteria were unstained due to the inability of antibodies to penetrate cell membranes.

## Results

### MOI 40 and 30-min incubation are optimal conditions for significantly improved positive-to-negative control ratio in infection

We selected the monocyte-derived THP-1 cells as the cell infection model because they have previously been used to mimic human macrophage infection by *N. gonorrhoeae*^12^. We differentiated THP-1 cells into macrophage-like cells (dTHP-1) and confirmed that dTHP-1 is a valid model for the evaluation of antibody-mediated opsono-phagocytic activity by quantifying the presence of Fc receptors CD64 and CD32 and of surface marker CD11b for macrophage differentiation via cytofluorometry (Supplementary Fig. 1). As previously reported by Auwerx and colleagues, expression of FcγRI and FcγRII decreased upon differentiation of THP-1 cells with phorbol 12-myristate 13-acetate (PMA)^16^.

**Figure 2:**
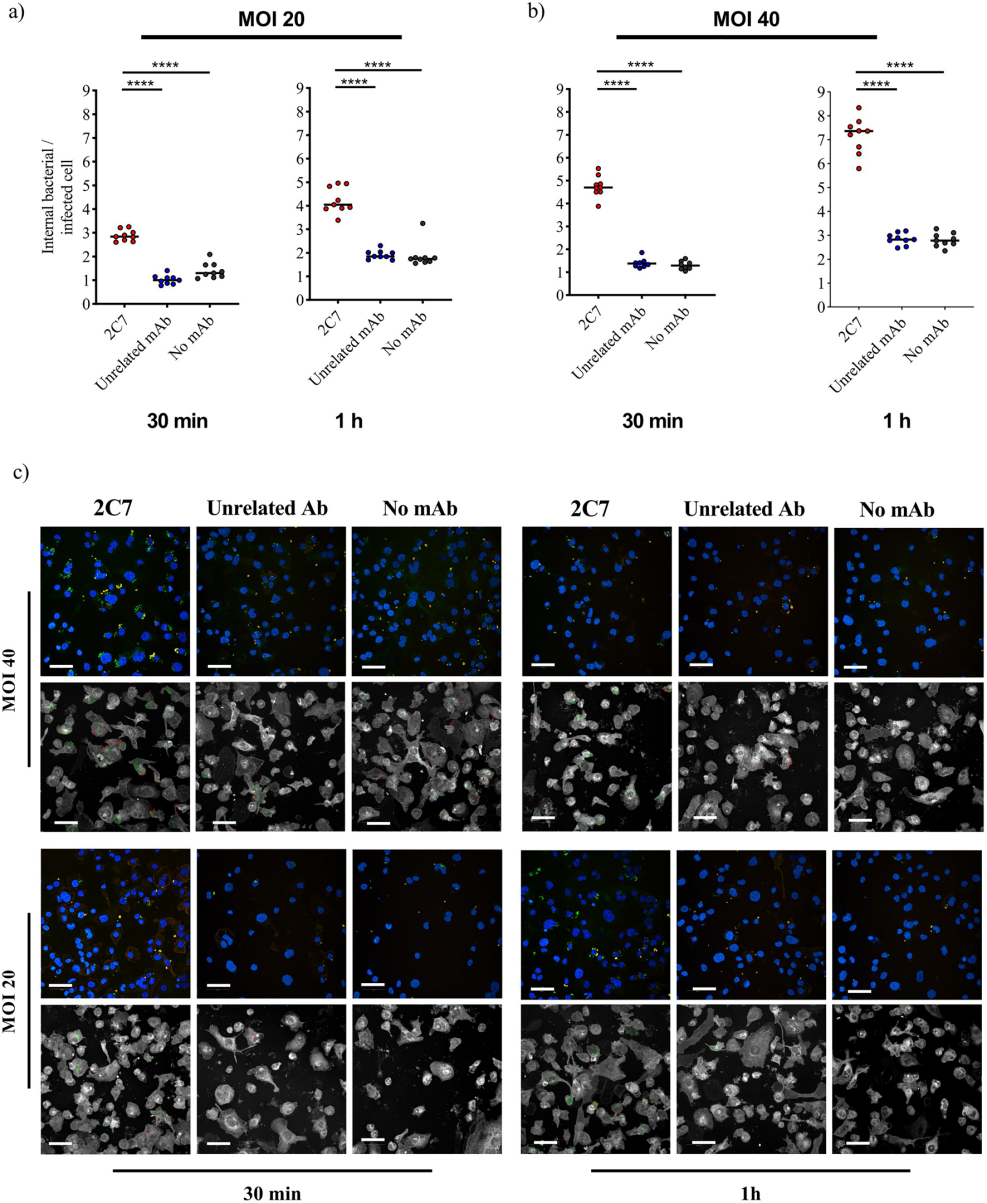
Evaluation of mAb activity at different MOIs and times of infection. The graphs report the number of internal bacteria per infected cell for the conditions MOI 20 (**a**) and MOI 40 (**b**), and at two different infection times, 30 min and 1-h. The 2C7 and the unrelated mAb were tested at a concentration of 10 μg/ml. For each condition 9 technical replicates were performed. The unpaired non-parametric t-test (Mann–Whitney test) p-values are reported on the graph. ****, p < 0.0001. (**c**) Representative images for each infection condition and treatment. The figure shows the number of internal bacteria per infected cell in two MOIs (MOI 20 and MOI 40) and two times of infection (30 min and 1-h conditions). The CellMask staining was removed for better visual inspection. Each block reports acquired images on top, and analysed images below. In the analysed images the internal and external bacteria are marked in green and red respectively. Scale bar is 50 μm.

To study antibody mediated enhancement of phagocytosis in vitro, two factors must be calibrated: the number of bacteria used to infect the target cell line and the time of infection. The number of bacteria is conventionally quantified by the multiplicity of infection (MOI) as the ratio between the number of bacteria and the number of target cells present in the well. To select the best experimental conditions to observe antibody mediated phagocytosis, we developed an image analysis pipeline using the commercial high-content screening Harmony software (version 4.9, Revvity) to quantify the ratio between the total number of internalised bacteria and the total number of infected cells upon incubation for 30 min or 1 h with MOIs 20 (Fig. 2a and Supplementary Table 1) and 40 (Fig. 2b). Furthermore, to validate 2C7 mAb as a positive control for the assay, we compared the infection conditions in the presence of 2C7 and of an unrelated mAb (expressed as recombinant proteins) and in the absence of mAbs. We observed that, in the absence of mAbs, dTHP-1 cells engulfed FA1090, thus establishing a base-line value for opsonophagocytic levels, while 2C7 mAb was able to enhance the phagocytic activity of the cells. Figure 2c depicts the images used to quantify the internal bacteria per infected cell.

According to the data shown in Fig. 2a and in Table 1, we concluded that a 30-min infection with MOI 40 represented the optimal condition for the establishment of the assay, with a signal-to-noise ratio of 3.4 (number of internalised bacteria per infected cell for the positive control divided by the negative control). Infection times shorter than 30 min were not considered because the number of engulfed bacteria would be too low for detection and for statistically significant analyses. With these settings, we could effectively amplify the assay range and keep low variability levels.

**Table 1:** vOPA infection conditions.

MOI	Infection Time	mAb	Median	Std	Signal-to-noise ratio
20	30 min	2C7	2.84	0.22	1.0
		Unrelated	1.0	0.18	
20	1 h	2C7	4.04	0.52	2.38
		Unrelated	1.85	0.18	
**40**	**30 min**	2C7	4.7	0.44	**3.4**
		Unrelated	1.38	0.19	
40	1 h	2C7	7.36	0.72	2.6
		Unrelated	2.83	0.23	

Median and standard deviation for the number of internalised bacteria per infected cells for the 2C7 and Unrelated mAb conditions.

Values in bold represent the infection conditions chosen to carry out the assay.

### Deep learning defines a vOPA read-out, named Phagocytic score, that significantly differentiates positive and negative controls

**Figure 3:**
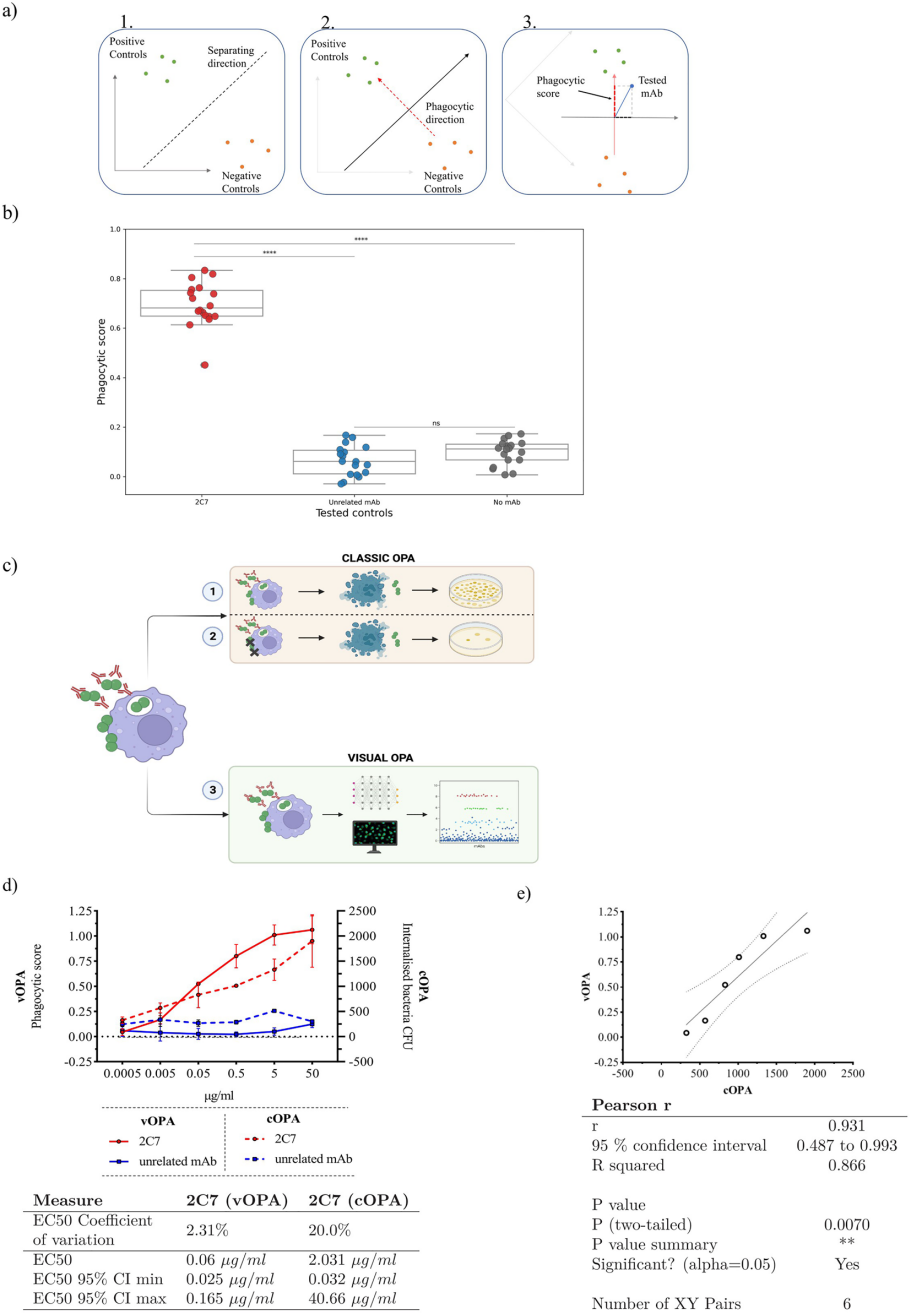
Validation of phagocytic activity promoted by 2C7 in vOPA. (**a**) Schematic representation of phagocytic score calculation using linear SVM on networks embedding. 1. We derived the separating direction (dashed line) of a binary classifier tasked to recognise positive and negative controls. 2. We computed the orthogonal direction with respect to the separating direction. This is the Phagocytic direction (red dashed line). 3. On testing a new mAb, indicated by the blue dot, we obtain the Phagocytic score by measuring the orthogonal projection on the phagocytic direction. (**b**) Phagocytic score calculated for 2C7, unrelated mAb and no mAb conditions using 18 technical replicates. The phagocytic score is reported on the y-axis. The unpaired non-parametric t-test (Mann–Whitney test) p-values are reported on the graph. ****, p < 0.0001; ns, not significant. (**c**) Graphical representation of the classical OPA (cOPA) and visual OPA (vOPA). cOPA measures the number of total and internalised bacteria through two approaches: (1) Total number of bacteria is determined by permeabilizing cells with saponin and releasing adherent and internalised bacteria. (2) Extracellular bacteria are killed by gentamicin, cells are permeabilized and internal bacteria are released. Both bacterial populations obtained in (1) and (2) are plated on agar plates and colonies are counted. The vOPA measurement is performed in one single step. After THP-1 infection, fluorescent dyes are used to stain bacteria and THP-1. Images generated with confocal microscopy are analysed to derive a phagocytic score to quantify mAb phagocytosis promoting activity. Image created with BioRender.com. (**d**) Comparison of results obtained with cOPA and vOPA using 2C7 and unrelated mAb tested in serial tenfold dilutions. The cOPA read-out is the number of internalised bacteria (CFU/well, right y axis), and vOPA read-out is the phagocytic score (left y axis) obtained with the CNN approach described in the methods section. Antibody concentrations are shown on the x axis. The underlying table reports the coefficient of variation for the EC50 values derived by fitting a three-parameter Hills curve to the 2C7 data. (**e**) Linear correlation for the 2C7 values measured in vOPA and cOPA. The values in the two assays are linearly correlated with a significant p-value (0.007).

Given the statistically significant difference reported in Fig. 2a, we used 2C7 and the unrelated antibody as positive and negative controls, respectively. To quantify mAb phagocytosis promoting activity, we adapted the image-analysis approach previously developed by Mascolini and colleagues^18^. In particular we used a convolutional neural network (CNN) to obtain a high-dimensional space to represent the images as feature vectors. This was obtained by fine-tuning a DenseNet161^15^ to classify positive versus. negative control images. The DenseNet last layer vectors, of dimension 2208, are projected in two-dimensional space by leveraging a soft margin linear SVM^18^. In particular, we obtained the first direction as the separating hyperplane of the linear SVM^18^, which discriminates between the positive and negative controls in the high-dimensional feature space. We have derived the second dimension as the direction orthogonal to the separating hyperplane, fitted by the linear SVM. This direction was named Phagocytic score. We summarised the adopted methodology in a schematic figure (Figure 3a1–3).

We tested positive and negative controls (2C7 and unrelated mAb) expressed as transcriptionally active PCR in supernatants and the sample without any mAb (no-mAb) with 18 replicates each. Figure 3b reports on the y axis the value of the Phagocytic score for the three conditions. The boxes representing median and quartiles of the corresponding distribution are visually non overlapping. Moreover, the Mann–Whitney p-value (p-value < 0.001) suggests that there is a significant difference between the distributions of the positive (2C7) and the negative control groups. vOPA was therefore considered as a robust assay to quantify antibody-mediated phagocytosis of *N. gonorrhoeae* by dTHP-1. Furthermore, we assessed the validity of vOPA as a single-point screening assay by computing the Signal window and Z’ performance measures^19^. The two metrics reported in Table 2 were in the recommended and acceptable range (as defined by Iversen and colleagues^19^) demonstrating that Phagocytic score allowed improved differentiation between positive and negative controls most likely because it was independent of the scientist’s evaluation and of the parameters that had to be selected in Harmony for efficient image analysis. Overall, this demonstrates that the vOPA assay can be used to screen mAbs for their phagocytosis-promoting activity in single-point dilution experiments.

**Table 2:** Separability performance measures for control groups.

Assay performance measures	Value	Acceptance Range	
Signal window	6.23	**Excellent**	> 2
		Acceptable	> 1
		Unacceptable	< 1
Z’ factor	0.3	Excellent	> 0.5
		Acceptable	0 < Z’ < 0.5
		Unacceptable	< 0
Signal	0.63		
2C7 std	0.09		
Unrelated mAb std	0.06		

Phagocytic score separation measured between positive (2C7) and negative (Unrelated mAb) controls.

### The vOPA read-out to describe phagocytic activity of cells is confirmed by CFU counts in classical assay (cOPA)

To validate the Phagocytic score obtained, we compared the read-out of vOPA with standard phagocytosis assay (cOPA), based on CFU counting (Fig. 3c). We quantified the 2C7 phagocytosis-promoting activity in two dose-dependent experiments (Fig. 3d). In one case we measured the mAb phagocytosis-promoting effect by analysing the images and deriving the Phagocytic score, while in the other case by counting the colonies of the internalised bacteria on agar plates. Importantly, results obtained by vOPA mirrored those generated by cOPA. However, when EC50 values were measured by fitting a three-parameter sigmoidal dose–response function, we observed that EC50 in vOPA was equal to 0.06 μg/ml while it was 2.031 μg/ml in cOPA. Considering the confidence intervals obtained for the estimates, the EC50 values for the two assays are largely compatible. It is worth noting that the confidence intervals associated with determining EC50 values through cOPA procedures appear substantially broader than their counterparts obtained employing vOPA approaches.

We verified that the vOPA Phagocytic score was linearly correlated with the cOPA CFU count by computing the Pearson correlation coefficient of the 2C7 values (Fig. 3e). We obtained an R squared value of 0.866 with a significant p-value of 0.007. We concluded that the Phagocytic score truly reflected the phagocytic activity of mAbs measured by conventional CFU counts.

### vOPA is applicable to the screening of anti-N. gonorrhoeae mAbs through single-point dilution experiments in a 96-well format

**Figure 4:**
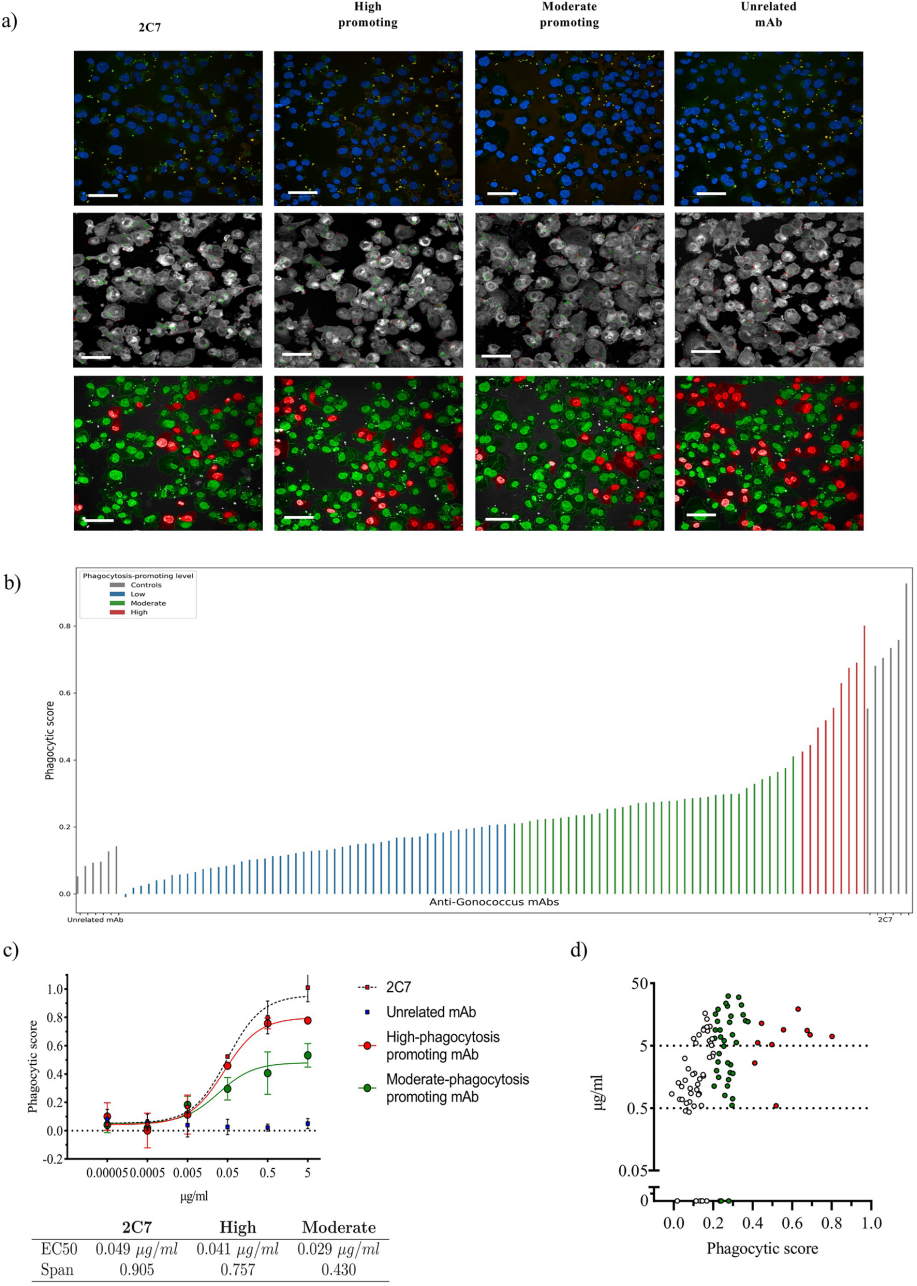
Application of vOPA to high-throughput screening and selection of positive-hits. **(a**) Images acquired in vOPA experiments performed using 2C7, high-phagocytosis promoting mAbs, moderate-phagocytosis promoting mAbs and negative control. The first row depicts the staining used in the assay (except for CellMask, for better visual inspection). The following two rows show two steps of the whole image analysis pipeline: the second row shows the image analysis step for the quantification of internal bacteria (green) and external bacteria (red), and the third row depicts the image analysis step which differentiates infected cells (green) and non-infected cells (red). Scale bar 50 μm. (**b**) HTS screening of 96 mAbs using vOPA. Each mAb was tested at one single dilution with no previous knowledge of the concentration. The phagocytic score is reported on the y-axis. The colours represent three phagocytosis-promoting activity groups. In blue no/low activity, in green moderate activity and in red high phagocytosis promoting activity, respectively. Results for the positive control (2C7) and negative control (unrelated monoclonal antibody) are depicted in grey. (**c**) Fitted dose-response curves of the positive control (2C7) and the mAbs selected from the screening of the 96 anti-*N. gonorrhoeae* mAbs. In red is reported the mAb from the high phagocytosis promoting group, and in green the mAb from the moderate phagocytosis promoting group. The negative control (unrelated mAb) is indicated by blue squares. mAb concentrations are on the x axis, while the number of internal bacteria per infected cell is on the y axis. The table shows EC50 and span values for 2C7, high-phagocytosis promoting and moderate-phagocytosis promoting mAbs. (**d**) Correlation between mAb concentration and phagocytic score with respect to the negative control. Concentrations are displayed on the y axis, while phagocytic score is reported on the x axis. The three colours represent the three groups of mAbs described in the text: white means no/low activity, green is for moderate activity and red indicates high phagocytosis promoting activity.

We leveraged the high-throughput image acquisition capability of the Opera Phenix system to screen an array of 96 human recombinantly expressed anti-FA1090 mAbs with unknown concentration and target for their ability to promote cell phagocytic activity against FA1090. Figure 4a exemplifies the different images obtained from experiments where one single mAb dilution was tested. These images were used to calculate the Phagocytic score for the 96 mAbs and 6 replicates of the positive and negative controls (Fig. 4b). Based on the assay signal, we separated the Phagocytic score range in three intervals of equal amplitude representing low-, moderate- and high-phagocytosis-promoting mAbs. Out of the 96 tested mAbs, 51% were low, 38% moderate and only 11% of them showed high-phagocytosis-promoting activity.

### The two potency classes “high-phagocytosis” and “moderate-phagocytosis” were confirmed in dose–response studies with purified mAbs

Based on the Phagocytic score we selected two candidate mAbs, one from the high-phagocytosis promoting group and one from the moderate phagocytosis-promoting group. Both mAbs were expressed, purified, and tested in a tenfold serial dilution experiment starting from a concentration of 5 μg/ml to confirm their activity. In Fig. 4c we reported the Phagocytic score values for the high- and moderate phagocytosis-promoting mAbs together with 2C7 and the unrelated control. We observed a concentration-dependent effect for the two selected mAbs and for 2C7 with the Phagocytic score values nicely fitting a 3-parameter sigmoidal dose–response curve which allowed calculation of EC50 and Span. EC50 for 2C7 was 0.049 μg/ml with a span equal to 0.905 whereas the mAbs belonging to the high- and moderate phagocytosis-promoting group reported an EC50 of 0.041 and 0.029 μg/ml, with span values of 0.757 and 0.430, respectively. Overall, the definition of two potency classes for the antibodies has been confirmed by the two dose–response curves derived for the high-phagocytosis and moderate-phagocytosis promoting mAbs. This demonstrates that the assay is sensitive enough to detect two different potency classes of antibodies.

### Variability in small-scale expression of mAbs does not affect the Phagocytic score, as it is the result of the antibody intrinsic potency

The concentration of the 96 expressed mAbs was measured and found to range between 0.3 to 50 μg/ml, which is compatible with the small-scale expression system used. Figure 4d correlates the concentration of each mAb with the Phagocytic score and illustrates that the Phagocytic score can discriminate active from inactive mAbs, as well as moderate- from high-phagocytosis-promoting mAbs, for concentrations as low as 0.05 μg/ml. In addition, Fig. 4d shows that 88 (92%) of the small-scale expressed mAbs were tested at a concentration higher than 0.5 μg/ml, thereby supporting the use of the Phagocytic score as a high-throughput screening indicator of mAb mediated phagocytosis activity.

We noted that the most effective antibodies were not necessarily the ones that were expressed at higher concentration, as the majority of the moderately and highly active monoclonals were tested at a concentration around 10 μg/ml. This demonstrated that the phagocytosis-promoting activity measured via vOPA was not strictly driven by antibody concentration but instead reflected intrinsic antibody biological properties such as high affinity for their target antigens and cell surface receptors.

## Discussion

In this work we presented the development and validation of vOPA, an assay used to quantify the opsono-phagocytosis-promoting activity of mAbs against *N. gonorrhoeae* from fluorescence microscopy images. The staining panel, the infection protocol, the image acquisition strategy, and the image analysis pipeline were successfully optimised and used for single-point dilution screening of human mAbs in the 96-well format. Using vOPA in combination with an automated confocal microscopy platform, e.g. Opera Phenix, it is possible to screen 96 mAbs in a 96-well plate in one day, thus reducing the hands-on time and accelerating the discovery process.

Other works have previously used a conventional CFU counting approach^12^ or a more innovative FACS staining method^14^, but none was applied to the high-throughput capacity. To evaluate the functionality of a single antibody through CFU counting, a substantial time commitment is required. This can range from 1 to 2 h to plate the bacteria and 30 min to 1 h to count colonies on the following days, depending on the number of replicates and dilutions tested. Scaling up this procedure to accommodate 96 antibodies introduces additional challenges related to space constraints and increased risk of experimental errors. While one operator alone can perform the screening of 96 mAbs in vOPA, cOPA necessitates additional manpower.

Interestingly, when compared with the reference methodology based on CFU counting, vOPA demonstrated an almost 10 × reduction in the coefficient of variation for the EC50 of the 2C7 mAb, suggesting that, in terms of reproducibility, cOPA could result in values which differ among each other 10 times more than the values obtained with vOPA.

Furthermore, we demonstrated that vOPA is a specific assay. By performing the t-test, we did not measure any statistically significant difference between the phagocytic score obtained using the unrelated mAb and no mAb conditions. The control separability for the phagocytic score, as for the data reported in Table 2, is acceptable according to the assay performance measures and acceptance ranges described by Iversen and colleagues^19^.

The vOPA staining presented here is limited to four fluorescence channels (cell membrane, DNA, intracellular and extracellular bacteria). This approach does not provide additional information on the fate of engulfed bacteria. Since we rely on GFP to identify the total bacterial population, the assay is limited to a pathogen which can be transformed and reliably express the fluorescent protein homogeneously. Furthermore, image analysis was dependent on the existence of positive and negative controls. Without a good positive control, it would not be possible to accurately assign a phagocytic score to mAbs. Additionally, confocal microscopy requires cells and bacteria to be adherent to the imaging plate. Finally, given that the methodology is designed for high-throughput screening, appropriate storage and computational resources are required for efficient processing of raw images.

vOPA confirmed the previously documented enhancement of pathogen uptake mediated by the 2C7 mAb^11^. Moreover, the positive correlation between phagocytic score and CFU counts attests to the assay's reliability. While this procedure introduces substantial progress in the field, delving into the contributions of individual channels to the final Phagocytic score could enhance our understanding of the relevance of the staining method employed and may even simplify the labelling process.

In summary, vOPA serves as a milestone for advanced high-throughput investigations into the efficacy of mAbs against bacterial pathogens. Given the feasibility of the staining protocol and the utilisation of a deep-learning approach that overcomes limitations imposed by traditional segmentation methods, we believe that the assay can be easily extended to other bacterial species.

## Methods

### Cell cultures

Human monocytic leukaemia cell line THP-1 (ATCC, Manassas, Virginia, USA) was cultured in RPMI1640 with Glutamax (Gibco), 10% fetal bovine serum (Gibco), 1 mM sodium pyruvate (Gibco) and 10 mM Hepes (Gibco) at 37 °C and 5% CO_2_. Cells were passaged three times per week and kept at a density below 1 × 10^6^ per ml. Cells were stimulated for three days with 30 nM phorbol-12-myristate-13-acetate (PMA) in 96-well plates (Cell Carrier 96 Ultra, Black, Clear bottom TC treated, cyclic olefin, Revvity) at the density of 40,000 cells / well. Culture medium was then removed and replaced with RPMI without PMA for the following 48 h.

### FACS analysis

Expression of IgG surface receptors and differentiation markers in THP-1 and differentiated THP-1 (dTHP-1) was tested by flow cytometry with the following reagents: CD64 PE-Cy7 1:200 and isotype mouse IgG1 k 1:50 (BD Pharmingen), CD32 BV711 1:30 and isotype mouse IgG2b k 1:30 (BD Pharmingen), CD11b BUV805 1:50 (BD Pharmingen), CD14 BV786 1:100 (BD Pharmingen). Cells were stained for CD45 BUV395 1:80 (BD Pharmingen). 2 × 10^5^ THP-1 cells per well were plated in a 96-well plate. According to the manufacturer’s protocol, cells were stained for LIVE/DEAD™ Fixable Near-IR Dead Cell Stain Kit as reported in the product datasheet (Thermo Fisher) and after blocking with rabbit serum, mAbs (25 μl) and respective isotypes were added and incubated for 20 min on ice and in the dark. After washing, Cytofix/Cytoperm (BD) was added and kept for 20 min on ice, followed by PermWash 1X (BD). Intracellular stain of CD68 PE-CF594 1:200 (BD Pharmingen) and its respective isotype mouse IgG2b k isotype 1:3333 (BD Pharmingen) followed for 20 min. Washes were performed with PermWash 1X and PBS. Acquisition was done at Fortessa (BD biosciences).

### Bacterial strains and culture conditions

*N. gonorrhoeae* strain FA1090 was used in this study and was cultured on gonococcal medium base agar (Difco™ GC Medium Base, BD) plus Isovitalex (BD). The strain was typically grown at 37 °C and 5% CO_2_ for approximately 15 h. The strain was engineered to express a superfolder green fluorescent protein (sfGFP)^20^ by integration of the encoding gene into the chromosome between genes NGO0077 and NGO0078, according to published protocols^21^. The modified strain is called FA1090::sfGFP. Prior to cell infection, bacteria were suspended in gonococcal liquid medium containing Isovitalex and grown at 37 °C to mid-logarithmic phase.

### Expression of mAbs into cell culture supernatants

The 96 mAbs with unknown target used in this work derive from a study conducted by Fondazione Toscana Life Sciences to identify anti-*N. gonorrhoeae* antibodies from patients vaccinated with an anti-meningococcal vaccine^22^.

Expression vectors encoding for anti-*N. gonorrhoeae* antibody heavy and light chains were used as templates for transcriptionally active PCR (TAP) reaction^23^. The resulting linear DNA fragments were used for transient transfection of the Expi293F cell line (Thermo Fisher Scientific) with a heavy:light chain ratio equal to 1:2. The transfection process lasted for six days at 37 °C with 8% CO2 in shaking conditions according to the manufacturer’s protocol (Thermo Fisher Scientific, US). Cell culture supernatants were harvested six days after transfection and clarified by centrifugation (4,500 × g, 15 min, 4 °C). The reference antibody 2C7 was expressed in Expi 293 cells as well. For medium-scale mAb expression and purification, expression vectors encoding for anti-*Neisseria gonorrhoeae* antibody heavy and light chains were used for transient transfection of Expi293F cells in a total volume of 60 ml. Antibodies were purified by affinity chromatography on protein G columns using an AKTA-Go system (GE Healthcare Life Sciences) as described below.

### Purification of mAbs by affinity chromatography

As previously reported by Andreano and collegueas^24^ filtered culture supernatants were purified by affinity chromatography. In details, supernatants were purified with a 1 mL HiTrap Protein G HP column (GE Healthcare Life Sciences) previously equilibrated in Buffer A (0.02 M NaH_2_PO_4_ pH 7). The flow rate for all steps of the HiTrap Protein G HP column was 1 mL/min. Culture supernatants were applied to 1 mL HiTrap Protein G HP column. The column was equilibrated in Buffer A for at least 6 column volumes (CV) which was collected as the column wash. Each monoclonal antibody was eluted from the column by applying a step elution of 6 CV of Buffer B (0.1 M glycine–HCl, pH 2.7) and by collecting elution fractions (1 ml). Eluted fractions were analysed by non-reducing SDS-PAGE and fractions showing the presence of IgG were pooled together. Final pools were dialyzed in PBS buffer pH 7.4 using Slide-A-Lyzer G2 Dialysis Cassette 3,5 K (Thermo Fisher Scientific) overnight at 4 °C. The dialysis buffer used was at least 200 times the volume of the sample. Antibody concentration was determined by measuring the absorbance at 520 nm using Pierce BCA Protein Assay Kit (Thermo Fisher Scientific). All the purified antibodies were aliquoted and stored at -80 °C.

### Quantitative enzyme linked immunosorbent assay (ELISA)

mAb concentration in collected supernatants was measured by quantitative ELISA in 384-well plates. Plates were pre-coated with goat anti-human IgG (2 µg/mL) (SouthernBiotech cat. N° 2040-01) and incubated at 4 °C. Blocking was performed with BSA 1%-PBS1X for 1 h at 37 °C followed by the addition of mAb supernatants diluted in BSA 1%-PBS1X-Tween 0.05%, initially 1:20 and then 1:2 for the following dilution steps. Human IgG-UNLB (SouthernBiotech cat. N° 0150-01) was used as a positive control (10 µg/mL). After 1 h of incubation at 37 °C, mAbs were washed away, a secondary goat anti-human IgG-Alkaline phosphatase (SouthernBiotech) antibody was added diluted 1:15,000 in PBS1X-BSA 1% + 0,05% tween for 1 h at 37 °C. The alkaline phosphatase substrate p-Nitrophenyl Phosphate (PNPP) (Sigma-Aldrich) was added and the reaction was incubated for 30 min at RT before the luminescence signal was read using a Varioskan™ LUX multimode microplate reader (Thermo Fisher Scientific, Waltham, MA, USA).

### Gentamicin protection assay (classical OPA assay)

THP-1 cells were seeded in 96-well tissue culture plates at 40,000 cells /well and subjected to the differentiation protocol as described above. Infection was performed as described above. As described by Château and colleagues^12^ to quantify the number of internalised bacteria, extracellular bacteria were washed away three times with RPMI medium and gentamicin (100 μg/ml) was added for 30 min to kill adherent bacteria. Cells were lysed with 0.5% saponin for 5 min and dilutions of the suspension containing bacteria were plated on GC agar. The number of CFU was determined after 24–48 h incubation.

### vOPA assay: plating and staining protocol

As previously reported by Maes and colleagues^25^, cells were seeded in 96 well plates, bacteria were stained with a secondary mAb and cells membrane and nuclei were stained with specific markers. Specifically, THP-1 cells were seeded and differentiated into 96-well plates as described above. After 5 days of differentiation cells were infected with FA1090::sfGFP grown to mid-logarithmic phase and pre-incubated with mAb supernatants diluted 1:5 in RPMI medium. After 30 min of pre-incubation, the mixture composed of mAbs and bacteria was added onto dTHP-1 at MOI 40. To synchronise the infection, the 96-well plates were centrifuged for 1 min at 200 xg. After 30 min of infection, each well was fixed with 2% paraformaldehyde (PFA) for 15 min, blocked with 1% (w/v) BSA. Extracellular FA1090::sfGFP bacteria were stained with primary antibody 2C7 (3 mg/ml) , for 1 h at RT, followed by secondary goat anti-Human IgG Alexa Fluor 568 (Thermo Fisher) diluted 1:2,000 at RT for 30 min. CellMask™Deep Red stain (Invitrogen) was used to stain the cell membrane, providing a means to delineate the cell boundary, and DAPI to stain cell nuclei and bacterial DNA.

### Confocal microscopy image acquisition

96-well plates were imaged with the microscope Opera Phenix High-Content Screening System (Revvity) using the 40 × objective, numerical aperture 1.1, acquiring 16 fields of view per well, and 13 images on the vertical dimension to form a z-stack per each field of view.

### vOPA image dataset

The vOPA image dataset consists of 9,048 4-channel images generated in 13 different experiments performed in 96-well imaging plates. A single dataset image corresponds to the maximum projection performed over 13 images acquired on the vertical dimension for all the fields of view. The raw images with full resolution and original colour depth (one TIFF file per channel, 16-bit grayscale, lossless compression) have a shape of 2,160 × 2,160 pixels (px) and were transformed into 8-bit images of size 512 × 512 to be processed with the convolutional neural network (CNN) and derive the corresponding phagocytic score by the CNN.

### Deep learning model

We downloaded and fine-tuned a DenseNet ^15^ model from the pytorch^26^ hub repository. The fine-tuning was performed as a binary classification task on the images acquired at the Opera Phenix, where the two classes were represented by cropped images from positive and negative controls respectively. To process the 4-channels vOPA images, we added a convolutional layer at the top of the pre-trained model. As optimizer we used SGD with 0.001 learning-rate and 0.9 momentum. We trained the model for 15 epochs. No hyperparameter optimization was performed. For the training we used 1 NVIDIA A100 40 GB GPU. To increase the amount of training data for our DenseNet model, we employed a data augmentation technique that involves cropping the input image. Specifically, we used a patch size of one half and a stride of one fourth of the input image respectively, generating with this process nine cropped images. These augmented images were then used to fine-tune the DenseNet instance.

### Harmony software image analysis

As previously reported by Maes and colleagues^25^, predefined building blocks in Harmony High-Content Imaging and Analysis Software version 4.9 (Revvity) were used to segment nuclei and cytoplasm in cells and count the individual bacteria. Moreover, we have also quantified the ratio between the total number of internalised bacteria and the total number of infected cells. Specifically, dTHP-1 cells were detected by analysing the combined signal of DAPI (nuclei) and CellMask (membrane), which allowed identification and counting of the two compartments. Then bacteria were localised and segmented by combining the DAPI and GFP signals. Bacteria that overlapped with the cytoplasm of a cell were identified as infecting and classified as internalised if they were negative for the immunostaining or adherent if they were positive for the immunostaining. Ultimately, cells with infecting bacteria were considered as infected cells. The complete analysis pipeline is reported in Supplementary Table 1.

### Data analysis

Statistical analysis, including Pearson correlation analysis and unpaired nonparametric t-test (Mann–Whitney test), was performed with GraphPad Prism 8 and the SciPy python library.

### Supplementary Information

Supplementary Information.

## Data Availability

The code was implemented in Python 3.8.13, pytorch version 1.8.2, sklearn version 1.0.2 and the TorchVision 0.10.0 models release. Our code is available at https://github.com/dasch-lab/vOPA.
