# Supplementary material for: Deep-learning image analysis for high-throughput screening of opsono-phagocytosis-promoting monoclonal antibodies against Neisseria gonorrhoeae
Source: Sci Rep. 2024 Feb 27;14:4807. doi: 10.1038/s41598-024-55606-4 (PMC10899611; doi:10.1038/s41598-024-55606-4)
Supplement: Supplementary file 1 — Supplementary Information. [file 41598_2024_55606_MOESM1_ESM.pdf]

Supplementary material

Supplementary Figure 1. **Evaluation of the presence of markers CD64, CD32 and CD11b in THP-1 and dTHP-1 using flow cytometry**. Numbers in each square indicate the percentage of THP-1 cells positive for the marker. The red colour intensity increases with the percentage of cells positive for the marker.

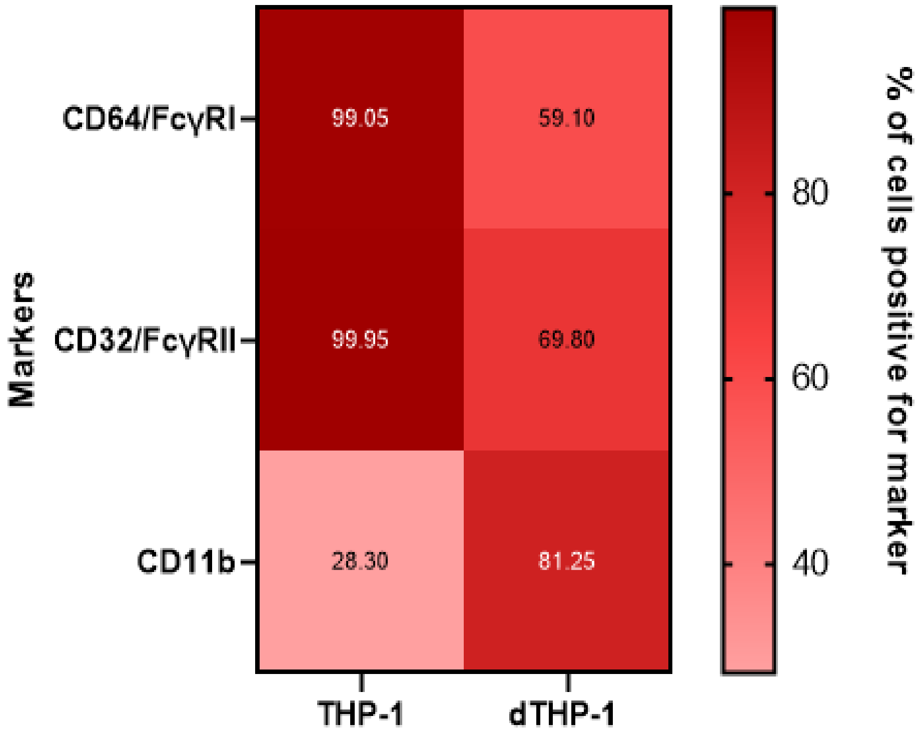

Supplementary Table 1. **Image analysis pipelines to count the number of internal bacteria per infected cell (Software: Harmony v4.9, Revvity).**

**Building Block**	**Input**	**Method**	**Output**	**Property prefix**
Calculate Image		Method:<br>by formula<br>Formula: A-300<br>A: DAPI<br>Negative values: Set to Zero<br>Undefined values:<br>Set to local average	Calculated Image	
Find nuclei<br><br>image ROI: none	Channel: Calculated<br>Common threshold: 0.41<br>Area:>30 um^2<br>Splitting coefficient: 7.0<br>Individual threshold: 0.39<br>Contrast:>0.11	Method: C	Nuclei	
Find Cytoplasm	Channel: CellMask Deep Red<br>Nuclei: Nuclei	Method: D<br>Individual threshold: 0,29		
Find Spots	Channel: EGFP<br>ROI: none	Method: C<br>Radius: <= 5,8 px<br>Contrast:>0.17<br>Uncorrected Spot to Region	Spots	

		Intensity> 1.3<br>Distance >= 1px<br>Spot Peak<br>Radius: 0 px<br>Calculate spot properties: yes		
Calculate Intensity Properties	Channel: DAPI<br>Population: Spots<br>Region: Spot	Method: Standard<br>Mean: yes<br>Maximum: yes<br>Quantile Fraction: 50%		Intensity Spot DAPI
Select population	Population: Spots	Method: Filter by property Intensity Spot DAPI mean:<br>>300<br>Spot area [px^2]:<br>>20<br>Boolean Operations: F1 and F2	Spots selected	Select population
Select population	Population: Spot selected	Method:<br>Select by Mask<br>Region: Spot<br>Mask population:<br>Nuclei<br>Mask Region: Cell Resized<br>Select by: Overlap>50%<br>Use inverted Mask:yes	Bacteria non-cytoplasm	

Select population	Population: Spot selected	Method: Select by Mask<br>Region: Spot<br>Mask population:<br>Nuclei<br>Mask Region:<br>Cell Resized<br>Select by:Overlap>50%<br>Use inverted Mask: no	Bacteria cytoplasm	
Calculate Intensity Properties	Channel: Alexa 568<br>Population: Spot selected<br>Region: Spot	Method: Standard<br>Mean: yes<br>Sum: yes<br>Maximum: yes Quantile Fraction: 50%		Intensity spot Alexa 568
Select population	Population: Spot selected	Method:<br>Filter by property<br>Intensity Spot Alexa 568 Mean:>500	Bacteria 568	
Select population	Population: Bacteria cytoplasm	Method: Select by Mask<br>Region: Spot<br>Mask population:<br>Bacteria 568<br>Mask region: Spot<br>Select by: Overlap > 50%	Adherent Bacteria	

		Use Inverted Mask:no		
Select population	Population: Bacteria cytoplasm	Method:<br>Select by Mask<br>Region: Spot<br>Mask population:<br>Bacteria 568 Mask region: Spot Select by: Overlap > 50% Use Inverted Mask: yes	Internal Bacteria	
Select population	Population: Bacteria non-cytoplasm	Method:<br>Select by Mask Region: Spot Mask population: Bacteria 568<br>Mask region: Spot<br>Select by: Overlap > 50% Use Inverted Mask: no	Non- adherent External Bacteria	
Calculate position properties	Population: Nuclei Region: Cell resized	Method: Cross-Population Population B: Spots Selected Region B:Spot Overlap:yes		Cell_with_bacteria
Calculate position properties	Population: Nuclei Region: Cell resized	Method: Cross-Population		Cell_with_ internal_bacteria

		Population B: Internal bacteria Region B: Spot Overlap: yes		
Select population	Population: Nuclei	Method:<br><br>Filter by Property Cell_with_ bacteria:>0	Infected cells	
Select population	Population: Nuclei	Method:<br><br>Filter by Property<br><br>Cell_with_ internal_ bacteria:>0	Infected cells with internalized bacteria	
